# Is AI Food a Gimmick or the Future Direction of Food Production?—Predicting Consumers’ Willingness to Buy AI Food Based on Cognitive Trust and Affective Trust

**DOI:** 10.3390/foods13182983

**Published:** 2024-09-20

**Authors:** Tiansheng Xia, Xiaoqi Shen, Linli Li

**Affiliations:** School of Art and Design, Guangdong University of Technology, Guangzhou 510090, China

**Keywords:** AI food, cognitive trust, affective trust, consumer acceptance, PLS-SEM

## Abstract

In recent years, artificial intelligence (AI) has been developing rapidly and has had a broad impact on the food industry, with food produced from AI-generated recipes already appearing to actually go on sale. However, people’s trust and willingness to purchase AI food are still unclear. This study builds an integrated theoretical model based on cognitive trust and affective trust, taking into account consumers’ quality value orientations, social norms, and perceived risks of AI food, with the aim of predicting and exploring consumers’ trust and acceptance of AI food. This study utilized the questionnaire method and 315 questionnaires were collected. The results of structural equation modeling (PLS-SEM) indicated that food quality orientation, subjective norms, perceived trust, and affective trust all had a significant positive effect on consumers’ purchase intentions. Perceived risk had a negative effect on affective trust and consequently on consumers’ purchase intention, but the effect on cognitive trust was not significant. The results also suggest that cognitive trust is the basis of affective trust and that consumer trust and acceptance of AI food can be enhanced by augmenting two antecedents of cognitive trust (food quality orientation and subjective norms). Possible practical implications and insights from the current findings are discussed.

## 1. Introduction

Although the rapid development of Artificial Intelligence (AI) has had a significant impact on various industries, the food industry has not really produced a high-value product with a stable customer base [1,2]. The application of AI in the food industry mainly stays at the level of packaging design, and even if AI is involved in many links such as design, production, and marketing [3], which can improve the efficiency of producers/service providers [4], for consumers, the existence of AI is unclear; the results are not significantly different from those of humans. And it seems that in many cases it only generates topic value [5]. The application of AI in existing products is gradually gaining attention, but these products or services are not always advertised to consumers. For instance, AI is employed in the dairy business for milk refrigeration [6], automated milking [7], and cheese quality inspection using computer vision systems [8]. AI technologies are employed in the beverage business, which includes both alcoholic and non-alcoholic beverages, to monitor the fermentation process [9], predict beverage quality [9], and assess the visual qualities of beverages using image analysis techniques [10]. AI is utilized in the bakery industry to automate the control of bread manufacturing, which includes parameter evaluation of the raw materials, dough quality control, and online or offline quality inspection of the finished product [11,12]. However, MUJI recently launched an AI French fries series [5]: through more than 3,000,000,000,000 simulations, recipes are selected from massive data, and unique flavored French fries made by balancing multiple ingredients are launched for the first time in the flagship store in Beijing, China. Its taste can cause discussion and controversy, and from the consumer’s perspective, it is an attempt to go very far in AI’s “intervention in design”. Moreover, some researchers also believe that there are opportunities for AI and machine learning in the food industry [13], where AI can significantly improve food packaging, extend shelf life, and ensure food safety by combining menus using AI algorithms as well as through the creation of more transparent supply chain management systems. Food design and formulation involves complex processes and many design criteria need to be taken into account when developing data-driven approaches; inverse design using deep generative networks is an emerging field that offers great potential for the design of novel food products [14]. Food firms can develop creative consumer-pleasing recipes with the use of artificial intelligence [15]. In a study on whether humans prefer diets designed by human nutritionists or AI [16], experts evaluated the composition, nutrition, and overall quality of diets designed by AI and human nutritionists. In a survey without menu name information, it was found that diets created by AI received more positive responses than nutritionist diets. This shows that AI has certain advantages and possibilities in diet design. Moreover, current research on consumers’ attitudes toward future food focuses more on discussing consumers’ acceptance, motivation, and continued willingness to purchase types of food such as green organic foods [17,18], plant-based meat substitutes [19], and genetically modified foods [20]. In existing research, more discussion has been focused on the technological development and potential of AI technology in the food field, and less attention has been paid to consumers’ trust and acceptance of AI’s involvement in the generation of future food recipes from a psychological perspective. In other words, although the application of AI technology in food design and production is becoming more and more mature, the academic and industry circles still have little understanding of how consumers view this change, especially their trust and attitude toward AI’s involvement in the generation of future food recipes. This research gap is particularly critical because it is directly related to whether AI food is accepted by the majority of consumers, and thus determines the application and development prospects of AI technology in the food industry.

This study used the questionnaire method and structural equation model to examine the factors and mechanisms that affect consumers’ willingness to purchase foods made from AI-generated recipes. It aims to explore consumers’ trust and acceptance of AI food, which refers to foods produced using recipes or formulas generated by AI technology, in order to understand the direction of AI food in future food production. The application of AI in the food industry is an emerging and rapidly developing field. Research on AI food is of great significance for understanding future food production trends and consumer attitudes. The research results can help food companies understand consumers’ attitudes and demands for AI food, thereby guiding product design, food production, and marketing strategies.

## 2. Literature Review and Hypothesis Development

The introduction and promotion of emerging food technologies is a complex process, which involves not only the safety and effectiveness of the technology itself but also the building of trust and psychological acceptance among consumers [21,22]. New food technologies are crucial to food safety and sustainability, but consumers are frequently hesitant to adopt them [23]. Previous studies [24,25] have shown that consumers’ attitudes and behaviors toward new food technologies are influenced and determined by multiple factors. It was demonstrated in a study on consumer acceptance of technology-based food innovations [21] that consumers’ willingness to accept innovative technologies is influenced by “proximal” determinants (perceived cost/benefit considerations, perception of risk and uncertainty, social norms, and perceived behavioral control) as well as by “distal” factors of acceptance (innovation characteristics, consumers, and social systems). It was noted that the acceptance of new foods is closely related to factors such as consumers’ personal values, cultural identity, and social belonging, in addition to the food itself (taste, price, and ease of preparation) in the investigation of how food neophobia affects consumers’ cognitive structure and willingness to accept new foods [22]. Furthermore, research has demonstrated [26] that food neophobia and food technology neophobia moderate the value-attitude-behavior model and that the attributes of 3D printed food—health, fun, creativity, and natural ingredients—play a significant and positive role in it. Although there are many factors influencing consumers’ attitudes and behaviors toward emerging food technologies, trust is a fundamental factor that permeates every stage of the decision-making process for consumers [27]. In addition to being a necessary condition for consumers to embrace new food technologies, trust is also a major motivator for changing consumer behavior [28]. We can gain more insight into how consumers consider and decide among a variety of influencing factors by conducting research on trust. The development of trust is a dynamic process involving the interaction of two levels: cognition and emotion [29]. In the promotion of food technology, consumers’ trust level will gradually develop from initial understanding of the technology to final acceptance as they acquire information, accumulate experience, and deepen their emotional connection. The promotion of emerging food technologies not only depends on the scientific verification and safety of the technology itself (i.e., cognitive trust) but also highly depends on consumers’ affective trust in technology providers. According to McAllister [30], there are two types of trust: affective trust and cognitive trust, which is particularly applicable to complex consumer behavior. This framework has been widely used in different research fields and contexts, such as service relationships (consumers and service providers) [31], entrepreneurship management [32], and trust in social robots [33], proving its cross-domain applicability.

**Figure 1:**
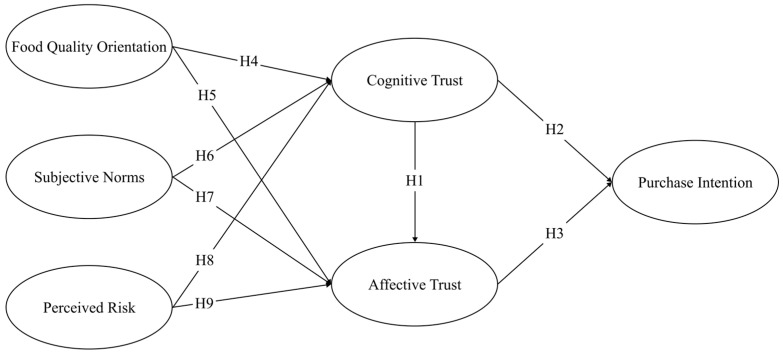
Research model. H means hypothesis.

Based on the theoretical framework proposed by McAllister [30], this study categorizes trust into two dimensions, cognitive trust and affective trust, as a way to deeply explore the impact of trust on consumers’ willingness to purchase AI food. Meanwhile, this study introduces three different perspectives and dimensions of food quality orientation, subjective norms, and perceived risk and uses them as antecedent variables of trust. By systematically examining the interactions between these latent variables, this study aims to reveal the relationship between trust and purchase intention and to explore the factors and mechanisms that influence consumers’ perceptions and acceptance of AI food. Figure 1 shows the conceptual research framework for consumer acceptance of AI food and the corresponding research hypotheses in this study (see Figure 1).

### 2.1. Cognitive Trust and Affective Trust

To understand consumer behavior under uncertainty, trust must be considered [34]. The concept of trust in technology is defined [35] as people’s assessment or anticipation of a technology’s ability to assist their work, based on its functionality, dependability, and usefulness. Meanwhile, McAllister [30] conceptualized trust as cognitive and affective perceptions. According to the theory of rational action [36], the concept of cognitive trust (COT) is consistent with the concept of trust belief, which refers to a principal’s rational expectation that the trustee will have the ability and capability to rely on. Cognitive trust stems from accumulated knowledge, which enables people to predict, with a certain degree of confidence, the likelihood that the technology can achieve its goals [37]. Although cognitive trust is knowledge-driven, it is predicated on an incomplete state of knowledge; in other words, if one is completely clear about how the technology or relationship will behave going forward, danger is removed and trust is unnecessary [38]. Affective trust (AFT), on the other hand, is defined as a trusting attitude [36] in which people feel safe and at ease depending on their cognitive trust in the trustee. It is distinguished by a feeling of safety and strength in the relationship. The essence of affective trust is the emotional reliance and confidence in technology or partners [38]. As the emotional bond becomes stronger, faith in it may grow beyond what is fair given the information that is currently available.

Our definition of cognitive and affective trust is consistent with the cognitive and affective core discussed above. Cognitive trust in this study refers to the trust formed by consumers in the safety, nutrition, and taste of AI food based on their existing rational thinking, analysis, and evaluation of empirical knowledge about AI technology, new foods, and other aspects. This kind of trust is based on consumers’ scientific knowledge, reliability understanding, and judgment of AI food. Affective trust refers to consumers’ emotional identification and preference for AI food. This kind of trust often stems from consumers’ emotional experiences with AI food, such as a sense of pleasure, satisfaction, and peace of mind. Previous studies [39,40] explored the relationship between cognitive trust and affective trust and showed that cognitive trust is positively correlated with affective trust, and cognitive trust provides a basis for affective trust. Moreover, cognitive trust and affective trust have been defined as trust beliefs and trust attitudes, respectively [41], which indicates that cognitive trust positively affects affective trust. What is more, trust is a dynamic accumulation process, and cognitive trust can be seen as the first step in the trust accumulation process. Over time, cognitive-based trust can be transformed into affective trust through repeated positive experiences [29]. This cumulative effect means that as consumers’ cognitive trust in AI food continues to increase, their affective trust will also grow. In terms of avoiding cognitive dissonance, people tend to maintain internal cognitive consistency and coherence [42]. In order to preserve this cognitive and emotional consistency, consumers who have a high degree of cognitive confidence in AI food will also be more likely to emotionally embrace and prefer these items in order to prevent the detrimental psychological experience of cognitive dissonance. Therefore, we assume that

**Hypothesis 1 (H1).**  *Cognitive trust is positively correlated with affective trust.*

In addition, trust plays a vital role in promoting consumers’ purchase intention (PI) and usage, including management, marketing, and information systems. Research from the past [20,43,44] has demonstrated the significance of trust in predicting people’s propensity to buy new foods, and generally speaking, trust is seen as a favorable result of “perceived probability”, “confidence”, and “expectation” [45]. Since AI intervention in the production of food recipes is a new approach and technology, it is very likely to develop personalized AI recipe customization. Determining how cognitive and affective trust interact can help to clarify how people make decisions about what to buy. It has been shown [35] that both cognitive trust and affective trust have a positive impact on continuance intention, that is, when consumers have higher levels of cognitive and affective trust, they are more inclined to continue to use and purchase services/products. In terms of cognitive trust, consumers’ expectations of product performance—which are grounded in logical analysis and scientific cognition—are raised by their cognitive trust in the safety, nutritional value, and flavor of AI foods. Customers are more inclined to buy a product when they are confident in its performance [46]. Regarding affective trust, customers are more likely to be inclined to buy when they experience favorable emotional reactions to AI food. Because affective trust is correlated with personal worth and happiness with the good or service, it can influence consumers’ willingness to buy [47]. Accordingly, we hypothesize that

**Hypothesis 2 (H2).**  *Cognitive trust is positively correlated with purchase intention.*

**Hypothesis 3 (H3).**  *Affective trust is positively correlated with purchase intention.*

### 2.2. Food Quality Orientation

Due to the recent spate of food safety issues, food quality is now one of the most crucial factors influencing consumers’ intentions to make a purchase [48]. Food quality orientation (FQO) is derived from the quality orientation category in the Food-Related Lifestyle Scale [49], which reflects consumers’ expectations of food quality and consists of six constructs, including natural/healthy, price/quality relationship, always new, organic, taste, and freshness. Expectations have been found to influence consumer acceptance of food [50]. In addition, the food quality orientation of this study is similar to the terminology of “perceived quality” in existing studies. According to Wang et al. [48], perceived quality encompasses both the inherent qualities of goods or services that draw customers in and eventually influence their purchase decisions, in addition to the subjective perceptions of those customers. Perceived quality is crucial among the factors that influence consumers’ willingness to purchase food, and studies [48,51] have shown that taste, nutritional value, and reliability are considered to be intrinsic indicators of perceived quality in food consumption research.

Furthermore, it is challenging to track the impact of novel foods and less likely for customers to keep buying and using them as they frequently make unsubstantiated claims about their sustainability, safety, or health benefits [21]. Thus, novel food design should focus on consumers’ food quality orientation to eliminate consumer anxiety and distrust. Food quality orientation affects consumer attitudes and behaviors because it is related to consumers’ perception of food quality. It has been shown that the quality of food innovations has a positive impact on consumer satisfaction [44] and that perceived quality attributes contribute to trust [52,53], both in terms of cognitive experience and trust, as well as in terms of emotional reliability and positive feelings. Therefore, we hypothesize that

**Hypothesis 4 (H4).**  *Food quality orientation is positively related to cognitive trust.*

**Hypothesis 5 (H5).**  *Food quality orientation is positively related to affective trust.*

### 2.3. Subjective Norms

According to definitions, subjective norms (SN) are those behaviors for which people feel pressured by society to comply or disobey [54]. Subjective norms can basically be considered as social norms (i.e., social pressure exerted by family, relatives, friends, leaders, experts, government, media, etc.) [55]. The theory of planned behavior [54] and the technology acceptance model [56] both contend that customers’ intents and behavioral results can be influenced by subjective norms, that is, they are important determinants of consumers’ intentions and behaviors to use and accept a product. Incorporating subjective norms into the study of consumers’ trust and acceptance of AI food is important and valuable because, in some cases, compared to people’s opinions regarding behavior, social pressure has a greater influence [57]. Moreover, subjective norms have been found to positively influence the consumption of various types of foods and beverages in existing consumer research areas, including consumer research areas on organic foods [57], green products [58], genetically modified foods [59], and novelty foods (insect products) [60].

It is important to note that a study conducted on the intention of consumers to adopt blockchain food traceability technology for organic food [61] discovered that subjective norms have a positive impact on trust, but the effect is not statistically significant. This finding contradicts the findings of numerous other types of research [55,62,63]. However, this does not imply that subjective norms have no role in the formation of consumer trust. Trust is a complex psychological state. On the contrary, it has shown that subjective norms can positively affect consumers’ willingness to trust [55]. Moreover, it has pointed out that subjective norms can affect individuals’ trust in food safety information [64]. In other words, if consumers perceive that important people around them trust a certain information source, this may increase their trust in the information source, that is, increase cognitive trust. Moreover, affective trust is an important component of the consumer–brand–product relationship, which involves consumers’ feelings of emotional attachment to and trust in brands and products. A recent study on food consumption [65] found that subjective norms can influence consumers’ brand preference for specific food products and that this emotional attachment can, in turn, enhance consumers’ trust and loyalty toward food brands. Based on the discussion above, we propose the following hypotheses:

**Hypothesis 6 (H6).**  *Subjective norms are positively related to perceived trust.*

**Hypothesis 7 (H7).**  *Subjective norms are positively related to affective trust.*

### 2.4. Perceived Risk

Perceived risk (PR) can be considered as an individual’s subjective view of the possibility of loss or risk based on his or her understanding and expectations of a situation or circumstance, which influences the individual’s decision-making process [55]. Perceived risk is usually considered negative [20], including unknown long-term effects, adverse health effects, and environmental and social issues [66]. There are many studies showing that consumers are cautious about new foods and related new food technologies. For example, genetic modification technology applied to food often generates high levels of perceived risk and aversion among consumers [67]. Moreover, the public’s perception of food safety is reflected in the perceived risk associated with foods containing additives [68]. Research on nanotechnology foods shows that the more positive attitudes one has toward trusting the institutions involved in using or regulating the technology, the less perceived risk one has, and that perceived risk is a strong predictor of the impacts associated with nanotechnology [23]. Additionally, research [24] has found that familiarity and understanding of food technology are some of the key factors that influence consumers’ reactions to new technologies. Deviations from traditional production methods tend to reduce the acceptability of food [69]. Consumers’ perception of unknown or uncontrollable risks in the food production process will negatively affect their trust in food technology. In other words, when consumers perceive unknown or uncontrollable risks and the perceived risks outweigh the benefits, they will be skeptical about the reliability and safety of the technology [70], thereby reducing cognitive trust. On the other hand, if consumers perceive higher risks, this may trigger negative emotions, weaken their trust in food technology and suppliers [71], increase their anxiety and worry, and thus reduce their affective trust [72]. Emotionally-based relational strength and a sense of security are hallmarks of affective trust. If consumers have higher perceived risks of new food technology, they may experience feelings of insecurity and anxiety [73]. In summary, it is hypothesized that

**Hypothesis 8 (H8).**  *Perceived risk is negatively related to cognitive trust.*

**Hypothesis 9 (H9).**  *Perceived risk is negatively related to affective trust.*

## 3. Methods

### 3.1. Participants

A convenient sampling method was used to distribute questionnaires to 377 potential respondents through an online questionnaire, and complete and valid data were received from 315 participants (83.55% of the respondents). The participants in this study were Chinese adult consumers from different cities in China. Among the participants, 69.21% were female and 30.79% were male. Table 1 shows the demographic information of the participants.

### 3.2. Instrument

The questionnaire for this study consists of three parts. The first part collects demographic information, namely gender, age, current city of residence, education level, marital status, personal monthly income, etc. The second part uses a five-point Likert scale with a score range of 1 (strongly disagree) to 5 (strongly agree) to measure Chinese consumers’ trust and acceptance of AI food by adapting previous research scales on latent variables (see Appendix A Table A1 for the questionnaire measurement items of this study). The third part is to add a trap question in the middle of the questionnaire item to measure the seriousness of the participants’ filling out, so as to facilitate the subsequent screening of valid questionnaires.

### 3.3. Procedure

The survey was carried out via an online questionnaire between April and June of 2024. To be more precise, the study team used Questionnaire Star (Changsha Ranxing Information Technology Co., Ltd. is located in Changsha, China.), the biggest online survey platform in China, to conduct an anonymous online survey. Questionnaire Star offers online questionnaire design and survey capabilities for businesses, research organizations, and people. It is comparable to Qualtrics, SurveyMonkey, or CloudResearch.

### 3.4. Data Analysis

In this study, the questionnaire was screened by taking into account the participants who chose to answer the trap questions incorrectly, as well as those who took too short or too long to complete the questionnaire. In order to examine the validity and reliability of the study and to test the overall study model, we employed partial least squares structural equation modeling (PLS-SEM) using SmartPLS (SmartPLS GmbH is located in Bönningstedt, Germany.) version 4.0 for data analysis. Partial least squares structural equation modeling (PLS-SEM), as opposed to covariance-based structural equation modeling (CB-SEM), can handle non-normal data and requires a smaller sample size [74]. In addition, because PLS-SEM is so good at resolving multicollinearity issues, it is a good choice for exploratory research and validating the created model structure [75]. In complicated study topics, it offers benefits for both exploratory and predictive analysis. Given the necessity to investigate intricate structural interactions and the relatively small number of participants (315), as well as the non-normal distribution of the data in this study [76], the PLS-SEM model was selected.

## 4. Results

### 4.1. Result of the Measurement Model

This study conducted an analysis of internal consistency, reliability, convergent validity, and discriminant validity on the measurement model. The measurement model was tested using composite reliability (CR), external loadings, and average variance extracted (AVE). To confirm the validity and reliability of the constructs, tests for convergent and discriminant validity were conducted. External loadings, consistent reliability coefficient (rho_A), AVE, and CR scores were assessed to confirm convergent validity [77,78]. The recommended thresholds for external loadings should be 0.60 or above, rho_A and CR should be 0.70 or above, and AVE should be 0.50 or above [79]. During the analysis, two items from food quality orientation, two items from perceived risk, two items from affective trust, and two items from purchase intention were eliminated due to low external loadings. As shown in Table 2, every external loading rho_A, CR, and AVE score fell between the suggested bounds, indicating that the reliability and convergent validity of all constructs were acceptable.

Moreover, to establish discriminant validity, the discriminant validity of each construct was assessed using the heterogeneous trait-monomorphic trait (HTMT) ratio method, and the value of HTMT should be less than 0.9 [80,81]. Based on the results in Table 3, discriminant validity can be constructed based on the HTMT method, and all the values in the table are less than 0.9, showing sufficient discriminant validity.

### 4.2. Result of the Structural Model

#### 4.2.1. Evaluation of the Model’s Capacity for Explanation

The coefficient of determination (R^2^) and the cross-validated redundancy (Q^2^) are the primary metrics used in structural equation modeling to assess a model’s explanatory capacity [82,83]. R^2^ measures the degree of explanation of the endogenous latent variables, and its explanatory power is indicated by values of 0.19–0.33, 0.33–0.67, and above 0.67, in that order [84]. The predictive relevance of the model is represented by Q^2^, and it has been observed that high model relevance is indicated by values of Q^2^ larger than 0 [82]. In Table 4, the explanatory power of the endogenous latent variables in this study is within a moderate range, which is acceptable. Additionally, all of the Q^2^ values are greater than 0, showing that the exogenous and endogenous factors have a significant predictive association.

#### 4.2.2. Testing Path Coefficient

**Figure 2:**
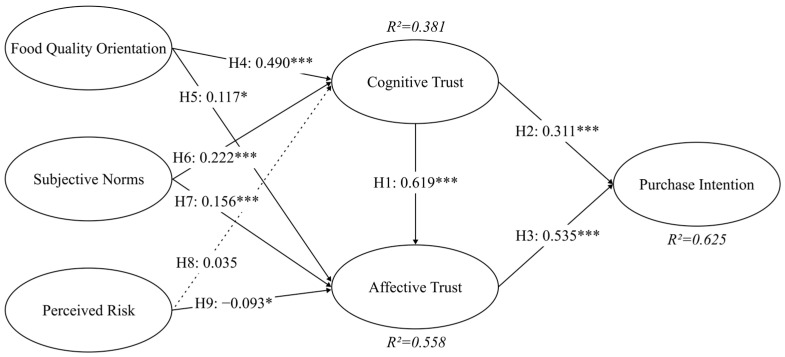
Structural model, * *p* < 0.05, *** *p* < 0.001. Dash line means not significant.

The raw data were repeatedly sampled and replaced using the partial least squares bootstrap method, and the T-statistic was used to determine the significance of each path coefficient. For the suggested research model, we present the standardized route coefficients and their findings in Table 5. The test results showed that eight hypotheses were confirmed (see the structural model in Figure 2). Consumers’ cognitive trust had a strong significant positive effect on both affective trust (β = 0.619, *p* < 0.001) and purchase intention (β = 0.311, *p* < 0.001), supporting H1 and H2. Affective trust had a strong significant positive effect on purchase intention (β = 0.535, *p* < 0.001), supporting H3. Food quality orientation has a significant positive correlation with cognitive trust (β = 0.490, *p* < 0.001) and affective trust (β = 0.117, *p* < 0.05), supporting H4 and H5. Subjective norms had a strong significant positive effect on cognitive trust (β = 0.222, *p* < 0.001) and affective trust (β = 0.156, *p* < 0.001), supporting H6 and H7. Perceived risk had no effect on cognitive trust, but was negatively correlated with affective trust (β = −0.093, *p* < 0.05). So H8 was not supported, but H9 was supported.

### 4.3. Testing Mediating Effects

This research uses the Bootstrapping resampling method to test the mediating impact in the model. The bias correction method is used to test the significance of the 95% confidence interval, and the confidence interval is found to not include 0 at either its upper or lower bounds. Table 6 displays the outcomes. We conclude that there is a strong mediation role played by both cognitive and affective trust.

## 5. Discussion

### 5.1. Interpretation of the Results

This study empirically tested the relationships among the determinants in the hypothesized model to investigate how these determinants explain consumers’ purchase intentions for AI food. The consumer acceptance model for AI food in this study identified the relationships among food quality orientation, subjective norms, perceived risk, cognitive trust, affective trust, and purchase intention. This model was tested using survey data from China. The results supported most of the hypotheses, but one hypothesis was not supported.

H1, H2, and H3 are valid, indicating that consumers’ cognitive trust in AI food positively affects affective trust, and cognitive trust and affective trust are important positive factors for purchase intention. These results corroborate previous research [35,39,85] and highlight the significance of winning over customers’ trust [43]. In the context of this study, AI food may be unfamiliar to consumers. To promote consumers’ acceptance and purchase of AI food, it is necessary to gain consumer trust. Cognitive trust is the basis of affective trust, which means that when consumers have a high degree of cognitive trust in AI food, this rational cognitive trust will be transformed into positive emotions, thus increasing affective trust. It shows that consumers must first have a certain level of knowledge and understanding of AI food, and establish a reliable and safe trust belief in AI food, in order to promote consumers’ emotional identification and love of AI food, thereby prompting consumers to try to buy it.

H4 and H5 are valid, suggesting that food quality orientation has a positive impact on consumers’ cognitive trust and affective trust, where food quality orientation is strongly positively correlated with cognitive trust. The three items of food quality orientation represent three dimensions, namely quality reliability, nutritional value, and taste texture, indicating that the three dominant variables are convergent, which agrees with the results of existing research [48]. For new foods, consumers may be more concerned about taste, safety, and nutrition [44]. Therefore, the results show that when consumers believe that the quality reliability, nutritional value, and taste texture of AI foods meet or exceed their expectations, they are more likely to have cognitive trust in AI foods. This trust comes from the understanding and recognition of food attributes, functions, or characteristics. Moreover, when consumers are satisfied with the quality orientation of AI food, they will develop an emotional connection with the product and are more likely to trust and be loyal to AI food.

H6 and H7 are supported, and this result confirms previous research [55,64,65], indicating that subjective norms are an important antecedent of trust and have a significant positive impact on cognitive trust and affective trust. It shows that the people or organizations around consumers play a crucial role in their willingness to trust AI food. This result is consistent with existing studies [48,86]. Whether it is the attitudes and behaviors of family members, colleagues, and other consumers or the information disseminated by government agencies and the media, they are all important factors in forming consumers’ subjective norms. In other words, the views and perceptions of people or organizations around consumers (such as family members, friends, other consumers, experts, and the government) on AI food will affect consumers’ cognitive trust in AI food. Meanwhile, when consumers feel positive attitudes toward AI food from people or organizations they trust, this emotional support will be transformed into consumers’ affective trust in AI food.

H9 is valid, whereas H8 is not. It means that consumers’ perceived risk of AI food does not affect their cognitive trust, and the higher the perceived risk, the weaker their affective trust. This may be due to the fact that cognitive trust is more based on facts and data (such as technology maturity, safety test results, etc.) [37], while affective trust is more influenced by personal emotions and intuition [87]. On the other hand, cognitive trust may take a long time to accumulate positive information to build, while affective trust is more susceptible to immediate emotional fluctuations [88]. Therefore, consumers’ cognitive trust is not greatly affected by perceived risk because it is more affected by cognitive experience and facts. While perceived risk is directly related to an individual’s emotional response [89], when consumers perceive a higher risk, they may unconsciously form a defensive attitude. This attitude directly affects the formation of affective trust, rather than affecting cognitive trust through a logical reasoning process. Additionally, the relationship between perceived risk and cognitive trust may be moderated by individual differences (technology familiarity, risk preference, personality traits, etc.) [90] and cultural or social norms [91]. Future research could consider exploring potential moderating factors.

Comprehensive analysis shows that food quality orientation and subjective norms have a positive and significant impact on cognitive trust and affective trust. Cognitive trust and affective trust as mediating variables of the impact of these two factors (FQO and SN) on purchase intention are also positive and significant, that is, food quality orientation and subjective norms will affect purchase intention through cognitive and affective trust. This shows that food quality expectations, social norms, and food trust are very important for consumers’ acceptance and purchase intention. In addition, perceived risk has a direct impact on consumers’ emotions and has a negative impact on affective trust. Affective trust, as a mediating variable of the impact of perceived risk on purchase intention, also has a significant negative impact. It suggests that high perceived risk will reduce consumers’ positive trust attitudes and acceptance.

### 5.2. Implications for Research

The theoretical model is supported by data and is of great significance as a robust model of consumers’ trust and acceptance of AI food. Starting from the cognitive and emotional dimensions of trust, this study combines three latent variables, food quality orientation, subjective norms, and perceived risk, to construct a model of consumers’ trust and acceptance of new AI food, enriching the theoretical models in the field of food consumption psychology and marketing. This empirical foundation enhances the originality and innovation of this study by providing specific insights into consumers’ quality expectations, social norms, concerns, and trust in AI food. This article supplements theoretical support on food quality expectations and perceived quality, subjective norms, perceived risks, cognitive trust, affective trust, and purchase intention. In addition, this study focuses on consumers’ trust and attitude toward AI involvement in food production and initially explores the possibility of AI food technology entering the mass consumer market.

### 5.3. Implications for Practice

Several practical suggestions can be drawn from the research results on how to promote consumers’ trust and acceptance of AI food. First, trust is the focus of this study, and cognitive trust is the basis of affective trust, which means that consumers need to establish deep cognitive trust in AI food first. The results of this study show that the antecedents of cognitive trust are food quality orientation and subjective norms. Therefore, in terms of food quality orientation, in order to ensure consumers’ trust and satisfaction with AI food, this inspires food companies to meet consumers’ expectations of AI food, that is, in product design and food production, it is necessary to strengthen the quality management, taste design, and nutritional matching of AI food [92]. Moreover, companies need to enhance the transparency of scientific information such as the working principle of AI food technology, the production process of AI food, and the health and safety assessment. In this way, consumers can see and perceive that their needs are met, thereby enhancing consumers’ perceived quality and expectations of AI food [93], so as to enhance their cognitive trust and promote purchasing behavior.

Second, subjective norms have a positive impact on both cognitive trust and affective trust. This finding is of great significance to the marketing of AI food. It is recommended that when promoting AI food, we should actively seek to establish positive social norms and consumer group recognition. Government propaganda, expert evaluation opinions, and media communication can all be used as effective means to influence information and have a positive effect on consumers’ trust and attitudes [94]. In other words, government agencies can highlight the potential advantages of AI food through publicity and education activities, strengthen consumers’ scientific understanding of AI food, and thus increase trust. At the same time, food companies can invite authoritative institutions, food science experts, and nutritionists to evaluate and recognize AI food and use this as publicity to increase consumer recognition and trust. The behavior and attitudes of other consumers can also affect the overall market’s acceptance of AI food. Therefore, this inspires food companies to establish certain interactive and evaluation mechanisms and platforms so that consumers can share their experiences, feelings, and feedback on AI food. This not only allows other consumers to see their real experiences and feelings but also collects consumer voices (suggestions and feedback) for companies to iterate and optimize AI food services. Of course, in the course of implementation, it is inevitable that some unscrupulous merchants or malicious consumers will break the evaluation platform. This requires the relevant government agencies to regulate and judge. At the same time, the companies or the relevant departments can set up evaluation reward and punishment mechanisms to achieve relative fairness and justice.

Finally, the negative impact of perceived risk on affective trust inspires food companies to focus on reducing consumers’ risk perception when promoting AI food, as well as strengthening consumers’ emotional connection with brands and AI food [95], so as to improve consumers’ affective trust and promote loyalty and positive acceptance. A study [96] has found that consumers who are more attached to brands are more likely to consume new foods and are more willing to try new things. Therefore, when introducing AI foods, food companies should take full advantage of the brand effect to positively reduce consumers’ risk perception while ensuring food safety. In the early stages of promotion, they can target those who trust brands, seek novelty and intense experiences, and enhance their love, connection, and enthusiasm for brands and products by providing more contact with consumers. Moreover, a long-term accumulation of good information can help to develop perceived trust. Therefore, businesses must sustain a positive brand image over time and keep providing consumers with positive scientific information about their products (process certifications and expert endorsements, such as the design, production, and testing of AI foods) in order to address perceived risks and increase cognitive trust. It is worth mentioning that in terms of reducing consumer perceived risks, the company will undoubtedly face the possibility that some people may still have deep-rooted negative views of AI food. This suggests that the company can gradually promote it through small-scale pilot projects and combine interactive and evaluation platforms to allow more consumers to participate and enhance their trust. As well, the company needs to accumulate cognitive trust for a long time, and this process will inevitably result in a situation where a lot of energy is spent but it is difficult to see results in the short term. For this, the company can gradually establish cognitive trust by regularly updating the technical progress of AI food, publishing long-term monitoring data and success cases, and using a variety of marketing channels and methods for publicity and display to maintain popularity for a long time.

### 5.4. Limitation and Future Research

Although the current study found some significant results, it also has certain limitations. Firstly, for the survey subjects of this study, we did not limit the study to a specific geographical area in China. In future studies, different consumer groups can be classified and analyzed in different dimensions, such as geographical location, personality traits, income, etc., for more in-depth exploration. Secondly, only quantitative research methods were used in this study. In future research, it is recommended to combine qualitative methods such as interviews and focus groups for analysis, so as to have a more comprehensive and in-depth understanding of consumers’ trust and attitudes toward AI food. Thirdly, this study was based on an online survey, which limited the participation of people with low incomes, disadvantaged areas, or difficulties in Internet access. Therefore, it is recommended that in future studies, a variety of survey methods and channels can be set up, such as combining online and offline methods, to obtain a wider range of sample data. Fourthly, H8 of this study was not supported. Perceived risk and cognitive trust may be influenced by moderating factors such as individual characteristics and cultural or social norms. Future studies might look into these moderating variables. Finally, considering that the application of AI technology in the field of food recipe making is still in its infancy, the number of consumers who have tried AI food is limited, so this survey did not ask participants whether they have tried AI food. However, as technology continues to advance and mature, it is recommended that future research conducts in-depth exploration and comparative analysis of the attitudes and opinions of consumers who have tried AI food and those who have not tried it, which will help to more fully understand consumers’ attitudes toward the promotion of AI food technology and its continued development.

## 6. Conclusions

This study developed a comprehensive model based on cognitive trust and affective trust and took food quality orientation, subjective norms, and perceived risk as antecedents to explain and predict consumers’ willingness to purchase AI food. The findings support most of the hypotheses proposed in the current study, indicating the key role of cognitive trust and affective trust in consumers’ purchase intention for AI food. The result provides important insights into consumers’ trust and acceptance of AI intervention in food production and enriches theoretical research on the psychology of consumption and marketing of new foods. Specifically, this study reveals how food quality orientation, subjective norms, and perceived risk drive purchase intention by influencing cognitive trust and affective trust, demonstrating and emphasizing the importance of focusing on consumer trust in the promotion of new foods. Through these findings, companies can design and promote AI foods with more targets, further understand the consumer trust-building process, and thus improve market acceptance. The research results not only have practical guiding significance for food companies but also provide a reference for policymakers and government regulators in the good development and supervision of the industry.

It is important to recognize the study’s limitations. Firstly, the survey respondents’ geographic location was not limited in this study. Future research can analyze and classify consumer groups in different dimensions (including but not limited to geographical location). Secondly, this study only used quantitative methods, and it is suggested that future research could combine quantitative and qualitative methods. Thirdly, the online survey method of this study failed to cover low-income groups and those with poor technical conditions. Therefore, future research should explore a variety of survey methods and channels (such as combining online and offline). Fourthly, the results of this study failed to support H8, and there may be potential moderating variables between perceived risk and cognitive trust. Future research can further discuss and explore specific moderating factors to improve the model and theory. Lastly, there are still few consumers who have tried AI food, and the use of AI in food manufacturing is still in its infancy. To obtain a more comprehensive knowledge of consumers’ attitudes regarding the promotion of AI food, it is advised that future research compares the opinions of consumers who have tried and those who have not.

## Figures and Tables

**Table 1:** Demographic characteristics (*n* = 315).

	Items	Number	Percentage(%)
Gender	Male	97	30.79
	Female	218	69.21
Age	18–25 years old	252	80.00
	26–35 years old	29	9.21
	36–45 years old	20	6.35
	46 years old or above (The oldest participant was 67 years old)	14	4.44
Education Level	High School and Vocational Senior High School or below	32	10.16
	College Diploma or Undergraduates	224	71.11
	Postgraduate Research or above	59	18.73
Marital Status	Unmarried	276	87.62
	Married	39	12.38
Personal Monthly Income	CNY 4000 or below	205	65.08
	CNY 4000–6000	57	18.10
	CNY 6000–12,000	39	12.38
	CNY 12,000 or above	14	4.44
City of Residence	Guangdong	147	46.67
	Jiangsu	17	5.40
	Hunan	13	4.13
	Shandong	13	4.13
	Sichuan	13	4.13
	Hebei	12	3.81
	Henan	11	3.49
	Fujian	10	3.17
	Beijing	9	2.86
	Jiangxi	8	2.54
	Zhejiang	8	2.54
	Tianjin	6	1.90
	Hubei	5	1.59
	Shanxi	5	1.59
	Shanghai	5	1.59
	Heilongjiang	4	1.27
	Macau	3	0.95
	Yunnan	3	0.95
	Chongqing	3	0.95
	Guangxi	3	0.95
	Shaanxi	3	0.95
	Anhui	2	0.63
	Xinjiang	2	0.63
	Liaoning	2	0.63
	Neimenggu	2	0.63
	Hainan	2	0.63
	Guizhou	1	0.32
	Jilin	1	0.32
	Hong Kong	1	0.32
	Gansu	1	0.32

Note: CNY means Chinese Yuan, which is the legal currency of the People’s Republic of China.

**Table 2:** Reliability and convergent validity for the measurement model.

Constructs	Items	Loadings	rho_A	CR	AVE
Food Quality Orientation	FQO1	0.867	0.816	0.89	0.729
	FQO2	0.849			
	FQO3	0.845			
Subjective Norms	SN1	0.683	0.777	0.845	0.521
	SN2	0.753			
	SN3	0.727			
	SN4	0.725			
	SN5	0.719			
Perceived Risk	PR1	0.888	0.884	0.849	0.654
	PR2	0.827			
	PR3	0.700			
Cognitive Trust	COT1	0.832	0.897	0.921	0.701
	COT2	0.867			
	COT3	0.802			
	COT4	0.873			
	COT5	0.808			
Affective Trust	AFT1	0.876	0.863	0.916	0.784
	AFT2	0.905			
	AFT3	0.875			
Purchase Intention	PI1	0.858	0.877	0.922	0.797
	PI2	0.923			
	PI3	0.896			

**Table 3:** Discriminant validity using the HTMT ratio.

Constructs	FQO	SN	PR	COT	AFT	PI
FQO						
SN	0.433					
PR	0.313	0.450				
COT	0.675	0.480	0.269			
AFT	0.600	0.496	0.147	0.825		
PI	0.519	0.433	0.149	0.792	0.874	

**Table 4:** Fit quality and test for predictive correlation.

Constructs	R^2^	Q^2^
COT	0.381	0.259
AFT	0.558	0.428
PI	0.625	0.489

**Table 5:** Results of hypothesis testing.

Hypothesis	Standard β	T Statistics	95% Bias Corrected Confidence Interval	Supported
H1: COT → AFT	0.619	12.667 ***	[0.520, 0.712]	YES
H2: COT → PI	0.311	5.039 ***	[0.198, 0.439]	YES
H3: AFT → PI	0.535	8.201 ***	[0.389, 0.649]	YES
H4: FQO → COT	0.490	8.771 ***	[0.377, 0.598]	YES
H5: FQO → AFT	0.117	2.361 *	[0.024, 0.218]	YES
H6: SN → COT	0.222	4.026 ***	[0.108, 0.325]	YES
H7: SN → AFT	0.156	3.182 ***	[0.056, 0.251]	YES
H8: PR → COT	0.035	0.738	[−0.067, 0.119]	NO
H9: PR → AFT	−0.093	2.108 *	[−0.181, −0.011]	YES

* *p* < 0.05, *** *p* < 0.001.

**Table 6:** Mediating effect test.

Relation of Path	The Point Estimate	T-Value	95% Bias Corrected Confidence Interval
FQO → COT → AFT	0.304	7.184 ***	[0.228, 0.395]
FQO → COT → PI	0.153	4.495 ***	[0.094, 0.229]
FQO → AFT → PI	0.063	2.312 *	[0.015, 0.123]
FQO → COT → AFT → PI	0.162	5.127 ***	[0.108, 0.233]
SN → COT → AFT	0.137	3.835 ***	[0.068, 0.209]
SN → COT → PI	0.069	3.178 ***	[0.033, 0.122]
SN → AFT → PI	0.083	3.189 ***	[0.035, 0.138]
SN → COT → AFT → PI	0.074	3.298 ***	[0.034, 0.121]
PR → COT → AFT	0.022	0.739	[−0.041, 0.073]
PR → COT → PI	0.011	0.717	[−0.022, 0.039]
PR → AFT → PI	−0.050	2.038 *	[−0.101, −0.007]
PR → COT → AFT → PI	0.012	0.728	[−0.022, 0.040]
COT → AFT → PI	0.331	6.646 ***	[0.232, 0.430]

* *p* < 0.05, *** *p* < 0.001.

## Data Availability

The original contributions presented in the study are included in the article, further inquiries can be directed to the corresponding author.

## Appendix A

**Table A1:** Part II of the questionnaire.

Construct	Item
Food Quality Orientation, FQO	
FQO1	I hope AI food will have higher reliability and better quality [48].
FQO2	I think AI food should have better nutritional value [48].
FQO3	I want the AI food to taste and texture great [44,48].
Subjective Norms, SN	
SN1	Family, relatives, and friends have a large influence on my decision whether to purchase AI food [48,97].
SN2	Colleagues and bosses (classmates and teachers) have a large influence on my decision whether to purchase AI food [48,97].
SN3	Other consumers have a large influence on my decision whether to purchase AI food [48,97].
SN4	Experts and scholars have a large influence on my decision whether to purchase AI food [48,97].
SN5	Government agencies or media have a large influence on my decision whether to purchase AI food [48,97].
Perceived Risk, PR	
PR1	I’m afraid that AI-generated recipe technology will monitor my behavior and worry about collecting too much personal information [70].
PR2	I worry that people won’t be able to figure out how AI-generated recipe technology makes decisions [70].
PR3	I think it’s risky to move too quickly to new food technologies [69].
Cognitive Trust, COT	
COT1	Given the scientific algorithms of AI technology, I believe in the capabilities of AI-generated technology for recipe generation [30,38,85].
COT2	I believe that AI-generated technology can generate safe recipes for manufacturers to produce food [30,38,85].
COT3	I believe that AI foods provide me with personalized nutritional value [30,38,85].
COT4	I am confident in the ability of AI technology to generate personalized scientific recipes to help people eat healthily [30,38,85].
COT5	I can trust food produced by professional and reliable manufacturers using AI-generated recipes [30,38,85].
Affective Trust, AFT	
AFT1	If I choose to eat AI food, I will feel relieved [30,38,85].
AFT2	I am satisfied and love AI Food, I feel it responds to my needs [30,38,85].
AFT3	I have a sense of intimacy with AI Food, it is like my dietary consultant [30,38,85].
Purchase Intention, PI	
PI1	I plan to buy AI food in the future [48,58].
PI2	I have a strong desire to buy AI food in the near future [48,58].
PI3	In the future, I plan to buy AI food more often [48,58].
